# 2-(2*H*-Benzotriazol-2-yl)-6-[(diethyl­amino)meth­yl]-4-methyl­phenol

**DOI:** 10.1107/S1600536809036575

**Published:** 2009-09-16

**Authors:** Jia-Ying Li, Yi-Chang Liu, Chia-Her Lin, Bao-Tsan Ko

**Affiliations:** a Department of Chemistry, Chung Yuan Christian University, Chung-Li 320, Taiwan

## Abstract

In the title compound, C_18_H_22_N_4_O, the dihedral angle between the planes of the benzotriazol unit and the phenyl ring of the phen­oxy group is 6.4 (2)°. There is an intra­molecular O—H⋯N hydrogen bond between the phenol and benzotriazol groups.

## Related literature

For background to the applications of amino­phenolate zinc compounds in the catalytic ring-opening polymerization of cyclic esters, see: Ejfler *et al.* (2008 ▶); Williams *et al.* (2003 ▶). For related structures: see: Li *et al.* (2009 ▶); Liu *et al.* (2009 ▶); Tsai *et al.* (2009 ▶).
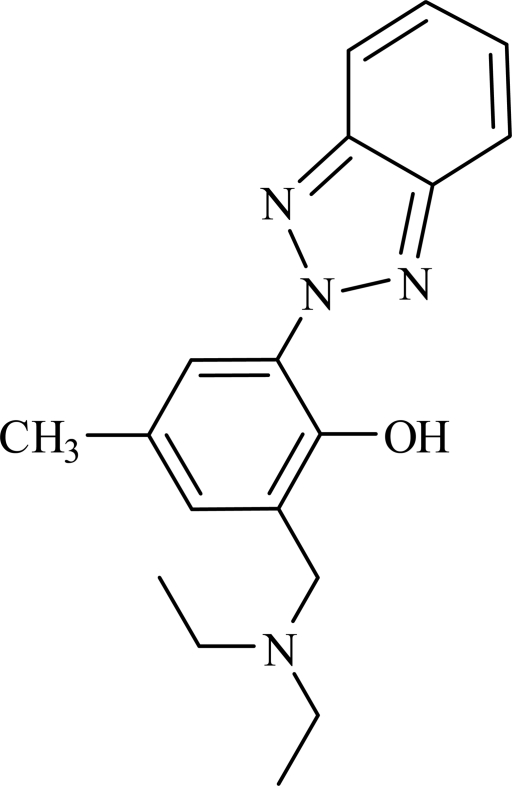


## Experimental

### 

#### Crystal data

C_18_H_22_N_4_O
*M* *_r_* = 310.40
Monoclinic, 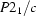

*a* = 8.3648 (4) Å
*b* = 20.0061 (8) Å
*c* = 10.0340 (4) Å
β = 100.200 (2)°
*V* = 1652.62 (12) Å^3^
*Z* = 4
Mo *K*α radiation
μ = 0.08 mm^−1^
*T* = 295 K
0.45 × 0.30 × 0.28 mm

#### Data collection

Bruker APEXII CCD diffractometer
Absorption correction: multi-scan (*SADABS*; Bruker, 2008 ▶) *T*_min_ = 0.972, *T*_max_ = 0.978
16312 measured reflections
3887 independent reflections
2643 reflections with *I* > 2σ(*I*)
*R*_int_ = 0.049

#### Refinement

*R*[*F* ^2^ > 2σ(*F* ^2^)] = 0.047
*wR*(*F* ^2^) = 0.123
*S* = 1.01
3887 reflections
209 parameters
H-atom parameters constrained
Δρ_max_ = 0.22 e Å^−3^
Δρ_min_ = −0.19 e Å^−3^

### 

Data collection: *APEX2* (Bruker, 2008 ▶); cell refinement: *SAINT* (Bruker, 2008 ▶); data reduction: *SAINT*; program(s) used to solve structure: *SHELXS97* (Sheldrick, 2008 ▶); program(s) used to refine structure: *SHELXL97* (Sheldrick, 2008 ▶); molecular graphics: *SHELXTL* (Sheldrick, 2008 ▶); software used to prepare material for publication: *SHELXTL*.

## Supplementary Material

Crystal structure: contains datablocks I. DOI: 10.1107/S1600536809036575/rk2166sup1.cif

Structure factors: contains datablocks I. DOI: 10.1107/S1600536809036575/rk2166Isup2.hkl

Additional supplementary materials:  crystallographic information; 3D view; checkCIF report

## Figures and Tables

**Table 1:** Hydrogen-bond geometry (Å, °)

*D*—H⋯*A*	*D*—H	H⋯*A*	*D*⋯*A*	*D*—H⋯*A*
O—H0⋯N1	0.82	1.90	2.621 (2)	146

**Fig. 1.**
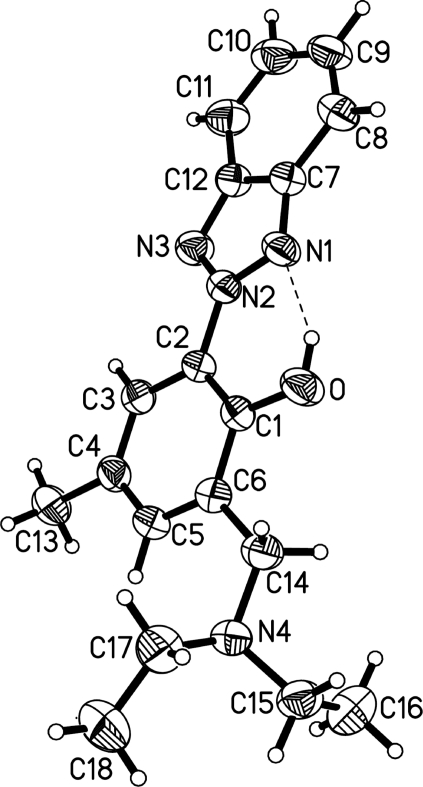
A view of the molecular structure of I with the atom numbering scheme. Displacement ellipsoids are drawn at the 50% probability level. H atoms are presented as a small spheres of arbitrary radius. Dashed line indicates the intramolecular hydrogen bond.

**Fig. 2.**
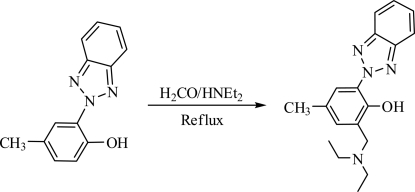
The synthesis path of I.

## supplementary crystallographic information

### Comment

Recently, amino-phenolate zinc compounds have been attracting considerable attention, mainly due to their applications in the catalytic ring-opening polymerization of cyclic esters (Ejfler *et al.*, 2008; Williams *et al.*, 2003). These amino-phenolate ligands were easily prepared by Mannich condensation from secondary amine, paraformaldehyde, and 2,4-di-substituted-phenol in the refluxing condition. Moreover, in terms of coordination chemistry, the additional amino group can provide the better chelation to stabilize the transition metal or main group metal complexes. Most recently, our group has successfully synthesized and structural characterized the Pd(II) and Al(III) complexes supported from 4-methyl-2-(2*H*-benzotriazol-2-yl)-phenolate (*BTP*) ligand (Li *et al.*, 2009; Tsai *et al.*, 2009). Therefore, our group is interested in the synthesis and preparation of amino-phenolate ligand derived from *BTP*-H. Herein, we report the synthesis and crystal structure of the title compound, (I), a potential ligand for the preparations of aluminium, palladium and zinc complexes (Scheme 1).

The molecular structure of I is composed of the benzotriazol-phenolate moiety and the diethylamino functionalized group (Fig. 1). The dihedral angle between the planes of the benzotriazol unit and the phenyl ring of the phenoxy group is 6.4 (2)°. There is an intramolecular O—H0···N1 hydrogen bond between the phenol and benzotriazol groups (Tab. 1). The distance of N1···H0 is substantially shorter, than the van der Waals distance of 2.75Å for the N and H distance. It is interesting to note that the six-member ring (O/C1/C2/N2/N1/H0) formed from the O—H···N hydrogen-bond is almost coplanar with the mean deviation of 0.016 (2)Å. Beside H-bonded motif, these bond distances of benzotriazol-phenolate group are similar to those found in the crystal structure of 2-(2*H*-benzotriazol-2-yl)-4-methylphenyl diphenylphosphinate (Liu *et al.*, 2009).

### Experimental

The title compound I was synthesized by the following procedures (Fig. 2): to a mixture of formaldehyde (3.60 g, 120.0 mmol) and diethylamine (12.53 ml, 120.0 mmol) was added 4-methyl-2-(2*H*-benzotriazol-2-yl)phenol (6.75 g, 30.0 mmol). The resulting mixture was heated under reflux for 2 day and then dried under reduced pressure to yield the oil residue. The residue was extracted with ethyl acetate (3 × 150 ml) and the organic layers were dried over MgSO_4_. The final solution was removed the solvent under vacuum to give white solids. Yield: 7.12 g (77%). Colourless crystals were obtained from the saturated hexane solution.

### Refinement

The H atoms were placed in idealized positions and constrained to ride on their parent atoms, with C—H = 0.93Å with *U*_iso_(H) = 1.2 *U*_eq_(C) for phenyl hydrogen; 0.96Å with *U*_iso_(H) = 1.5 *U*_eq_(C) for CH_3_ group; 0.97Å with *U*_iso_(H) = 1.2 *U*_eq_(C) for CH_2_ group; O—H = 0.82Å with *U*_iso_(H) = 1.5 *U*_eq_(O).

### Crystal data

**Table:**

C_18_H_22_N_4_O	*F*(000) = 664
*M**_r_* = 310.40	*D*_x_ = 1.247 Mg m^−^^3^
Monoclinic, *P*2_1_/*c*	Mo *K*α radiation, λ = 0.71073 Å
Hall symbol: -P 2ybc	Cell parameters from 5834 reflections
*a* = 8.3648 (4) Å	θ = 2.5–27.4°
*b* = 20.0061 (8) Å	µ = 0.08 mm^−^^1^
*c* = 10.0340 (4) Å	*T* = 295 K
β = 100.200 (2)°	Block, colourless
*V* = 1652.62 (12) Å^3^	0.45 × 0.30 × 0.28 mm
*Z* = 4	

### Data collection

**Table:**

Bruker APEXII CCD diffractometer	3887 independent reflections
Radiation source: fine–focus sealed tube	2643 reflections with *I* > 2σ(*I*)
graphite	*R*_int_ = 0.049
Detector resolution: 8.3333 pixels mm^-1^	θ_max_ = 28.3°, θ_min_ = 2.0°
φ– and ω–scans	*h* = −11→11
Absorption correction: multi-scan (*SADABS*; Bruker, 2008)	*k* = −26→26
*T*_min_ = 0.972, *T*_max_ = 0.978	*l* = −11→11
16312 measured reflections	

### Refinement

**Table:**

Refinement on *F*^2^	Secondary atom site location: difference Fourier map
Least-squares matrix: full	Hydrogen site location: inferred from neighbouring sites
*R*[*F*^2^ > 2σ(*F*^2^)] = 0.047	H-atom parameters constrained
*wR*(*F*^2^) = 0.123	*w* = 1/[σ^2^(*F*_o_^2^) + (0.045*P*)^2^ + 0.4268*P*] where *P* = (*F*_o_^2^ + 2*F*_c_^2^)/3
*S* = 1.01	(Δ/σ)_max_ < 0.001
3887 reflections	Δρ_max_ = 0.22 e Å^−^^3^
209 parameters	Δρ_min_ = −0.19 e Å^−^^3^
0 restraints	Extinction correction: *SHELXL*, Fc^*^=kFc[1+0.001xFc^2^λ^3^/sin(2θ)]^-1/4^
Primary atom site location: structure-invariant direct methods	Extinction coefficient: 0.0193 (16)

### Special details

**Table:**

Experimental. ^1^H NMR (CDCl_3_, ppm): δ 6.96–7.97 (6H, m, ArH), 3.83 (2H, s, –CH_2_NEt_2_), 2.64 (4H, q, –CH_2_CH_3_), 2.31 (3H, s, ArCH_3_), 1.08 (6H, t, –CH_2_CH_3_).
Geometry. All s.u.'s (except the s.u. in the dihedral angle between two l.s. planes) are estimated using the full covariance matrix. The cell s.u.'s are taken into account individually in the estimation of s.u.'s in distances, angles and torsion angles; correlations between s.u.'s in cell parameters are only used when they are defined by crystal symmetry. An approximate (isotropic) treatment of cell s.u.'s is used for estimating s.u.'s involving l.s. planes.
Refinement. Refinement of *F*^2^ against ALL reflections. The weighted *R*–factor *wR* and goodness of fit *S* are based on *F*^2^, conventional *R*–factors *R* are based on *F*, with *F* set to zero for negative *F*^2^. The threshold expression of *F*^2^ > σ(*F*^2^) is used only for calculating *R*–factors(gt) *etc*. and is not relevant to the choice of reflections for refinement. *R*–factors based on *F*^2^ are statistically about twice as large as those based on *F*, and *R*–factors based on ALL data will be even larger.

### Fractional atomic coordinates and isotropic or equivalent isotropic displacement parameters (Å^2^)

**Table:**

	*x*	*y*	*z*	*U*_iso_*/*U*_eq_	
O	0.38174 (14)	0.59067 (5)	0.55612 (11)	0.0515 (3)	
H0	0.4272	0.6187	0.6095	0.077*	
N1	0.45310 (16)	0.71135 (6)	0.64977 (13)	0.0428 (3)	
N2	0.35464 (15)	0.73474 (6)	0.53973 (12)	0.0370 (3)	
N3	0.35122 (17)	0.80050 (6)	0.52295 (13)	0.0431 (3)	
N4	0.12557 (16)	0.46827 (6)	0.25528 (13)	0.0450 (3)	
C1	0.27977 (18)	0.62176 (7)	0.45473 (14)	0.0372 (3)	
C2	0.26092 (18)	0.69109 (7)	0.44291 (14)	0.0359 (3)	
C3	0.15528 (18)	0.71937 (7)	0.33526 (15)	0.0387 (4)	
H3B	0.1442	0.7656	0.3294	0.046*	
C4	0.06657 (18)	0.67937 (8)	0.23680 (15)	0.0396 (4)	
C5	0.08776 (18)	0.61034 (8)	0.24786 (15)	0.0411 (4)	
H5A	0.0296	0.5831	0.1813	0.049*	
C6	0.19162 (19)	0.58095 (7)	0.35372 (15)	0.0389 (4)	
C7	0.52040 (19)	0.76738 (8)	0.71051 (15)	0.0399 (4)	
C8	0.6352 (2)	0.77626 (9)	0.83005 (17)	0.0519 (4)	
H8A	0.6783	0.7402	0.8829	0.062*	
C9	0.6802 (2)	0.84032 (9)	0.86450 (18)	0.0567 (5)	
H9A	0.7565	0.8478	0.9425	0.068*	
C10	0.6153 (2)	0.89575 (9)	0.78609 (18)	0.0564 (5)	
H10A	0.6494	0.9385	0.8143	0.068*	
C11	0.5046 (2)	0.88836 (8)	0.67066 (18)	0.0522 (4)	
H11A	0.4621	0.9250	0.6194	0.063*	
C12	0.45687 (19)	0.82249 (7)	0.63192 (15)	0.0399 (4)	
C13	−0.0484 (2)	0.70946 (9)	0.11941 (17)	0.0516 (4)	
H13A	−0.0474	0.7573	0.1280	0.077*	
H13B	−0.1563	0.6931	0.1194	0.077*	
H13C	−0.0147	0.6972	0.0361	0.077*	
C14	0.2234 (2)	0.50642 (7)	0.36353 (17)	0.0478 (4)	
H14A	0.3373	0.4984	0.3617	0.057*	
H14B	0.2014	0.4905	0.4498	0.057*	
C15	0.2099 (2)	0.40745 (8)	0.22420 (18)	0.0501 (4)	
H15A	0.1315	0.3767	0.1745	0.060*	
H15B	0.2586	0.3859	0.3082	0.060*	
C16	0.3396 (3)	0.42154 (9)	0.1423 (2)	0.0644 (5)	
H16A	0.3911	0.3804	0.1244	0.097*	
H16B	0.4190	0.4511	0.1919	0.097*	
H16C	0.2918	0.4421	0.0582	0.097*	
C17	−0.0343 (2)	0.45337 (10)	0.2867 (2)	0.0622 (5)	
H17A	−0.0713	0.4915	0.3328	0.075*	
H17B	−0.0251	0.4156	0.3482	0.075*	
C18	−0.1594 (3)	0.43745 (12)	0.1629 (3)	0.0840 (7)	
H18D	−0.2619	0.4283	0.1896	0.126*	
H18A	−0.1251	0.3990	0.1181	0.126*	
H18B	−0.1708	0.4750	0.1023	0.126*	

### Atomic displacement parameters (Å^2^)

**Table:**

	*U*^11^	*U*^22^	*U*^33^	*U*^12^	*U*^13^	*U*^23^
O	0.0601 (8)	0.0407 (6)	0.0447 (7)	0.0052 (5)	−0.0154 (5)	−0.0021 (5)
N1	0.0456 (8)	0.0430 (7)	0.0348 (7)	0.0023 (6)	−0.0065 (6)	−0.0028 (5)
N2	0.0374 (7)	0.0373 (6)	0.0339 (7)	0.0030 (5)	0.0000 (5)	−0.0027 (5)
N3	0.0497 (8)	0.0367 (6)	0.0401 (7)	0.0022 (6)	0.0006 (6)	−0.0014 (5)
N4	0.0456 (8)	0.0400 (7)	0.0465 (8)	−0.0009 (6)	0.0003 (6)	−0.0097 (6)
C1	0.0356 (8)	0.0410 (8)	0.0329 (8)	0.0047 (6)	−0.0002 (6)	−0.0020 (6)
C2	0.0343 (8)	0.0407 (7)	0.0310 (8)	0.0017 (6)	0.0015 (6)	−0.0056 (6)
C3	0.0393 (9)	0.0400 (8)	0.0351 (8)	0.0056 (6)	0.0022 (7)	−0.0012 (6)
C4	0.0345 (8)	0.0500 (9)	0.0326 (8)	0.0037 (6)	0.0017 (6)	−0.0009 (6)
C5	0.0389 (9)	0.0467 (8)	0.0351 (8)	−0.0004 (7)	−0.0007 (7)	−0.0076 (6)
C6	0.0378 (8)	0.0396 (8)	0.0378 (8)	0.0023 (6)	0.0030 (7)	−0.0047 (6)
C7	0.0382 (8)	0.0444 (8)	0.0360 (8)	0.0001 (6)	0.0034 (6)	−0.0060 (6)
C8	0.0514 (11)	0.0586 (10)	0.0408 (10)	0.0004 (8)	−0.0052 (8)	−0.0057 (7)
C9	0.0511 (11)	0.0698 (12)	0.0456 (10)	−0.0101 (9)	−0.0012 (8)	−0.0180 (8)
C10	0.0595 (12)	0.0536 (10)	0.0560 (11)	−0.0137 (8)	0.0103 (9)	−0.0167 (8)
C11	0.0610 (11)	0.0429 (8)	0.0518 (10)	−0.0053 (8)	0.0081 (9)	−0.0058 (7)
C12	0.0392 (8)	0.0433 (8)	0.0366 (8)	−0.0012 (6)	0.0053 (7)	−0.0052 (6)
C13	0.0492 (10)	0.0601 (10)	0.0403 (9)	0.0052 (8)	−0.0059 (8)	0.0024 (7)
C14	0.0519 (10)	0.0415 (8)	0.0449 (10)	0.0034 (7)	−0.0056 (8)	−0.0070 (7)
C15	0.0601 (11)	0.0379 (8)	0.0503 (10)	0.0008 (7)	0.0039 (8)	−0.0049 (7)
C16	0.0789 (15)	0.0567 (11)	0.0610 (12)	0.0135 (10)	0.0216 (11)	0.0026 (9)
C17	0.0537 (12)	0.0640 (12)	0.0686 (13)	−0.0041 (9)	0.0098 (10)	−0.0079 (9)
C18	0.0543 (13)	0.0847 (15)	0.1061 (19)	−0.0137 (11)	−0.0048 (12)	−0.0191 (13)

### Geometric parameters (Å, °)

**Table:**

O—C1	1.3572 (17)	C9—C10	1.412 (3)
O—H0	0.8200	C9—H9A	0.9300
N1—N2	1.3395 (16)	C10—C11	1.357 (2)
N1—C7	1.3503 (19)	C10—H10A	0.9300
N2—N3	1.3259 (16)	C11—C12	1.411 (2)
N2—C2	1.4323 (18)	C11—H11A	0.9300
N3—C12	1.3520 (19)	C13—H13A	0.9600
N4—C14	1.4548 (19)	C13—H13B	0.9600
N4—C17	1.458 (2)	C13—H13C	0.9600
N4—C15	1.4675 (19)	C14—H14A	0.9700
C1—C2	1.399 (2)	C14—H14B	0.9700
C1—C6	1.405 (2)	C15—C16	1.499 (3)
C2—C3	1.389 (2)	C15—H15A	0.9700
C3—C4	1.381 (2)	C15—H15B	0.9700
C3—H3B	0.9300	C16—H16A	0.9600
C4—C5	1.394 (2)	C16—H16B	0.9600
C4—C13	1.508 (2)	C16—H16C	0.9600
C5—C6	1.379 (2)	C17—C18	1.510 (3)
C5—H5A	0.9300	C17—H17A	0.9700
C6—C14	1.515 (2)	C17—H17B	0.9700
C7—C12	1.404 (2)	C18—H18D	0.9600
C7—C8	1.409 (2)	C18—H18A	0.9600
C8—C9	1.363 (2)	C18—H18B	0.9600
C8—H8A	0.9300		
			
C1—O—H0	109.5	C12—C11—H11A	121.5
N2—N1—C7	103.20 (12)	N3—C12—C7	109.07 (13)
N3—N2—N1	116.59 (11)	N3—C12—C11	129.73 (15)
N3—N2—C2	121.46 (12)	C7—C12—C11	121.20 (15)
N1—N2—C2	121.93 (12)	C4—C13—H13A	109.5
N2—N3—C12	102.94 (11)	C4—C13—H13B	109.5
C14—N4—C17	111.20 (14)	H13A—C13—H13B	109.5
C14—N4—C15	111.45 (13)	C4—C13—H13C	109.5
C17—N4—C15	111.75 (13)	H13A—C13—H13C	109.5
O—C1—C2	124.37 (13)	H13B—C13—H13C	109.5
O—C1—C6	117.02 (13)	N4—C14—C6	113.51 (13)
C2—C1—C6	118.58 (13)	N4—C14—H14A	108.9
C3—C2—C1	121.10 (13)	C6—C14—H14A	108.9
C3—C2—N2	118.39 (13)	N4—C14—H14B	108.9
C1—C2—N2	120.48 (12)	C6—C14—H14B	108.9
C4—C3—C2	120.50 (14)	H14A—C14—H14B	107.7
C4—C3—H3B	119.8	N4—C15—C16	112.49 (14)
C2—C3—H3B	119.8	N4—C15—H15A	109.1
C3—C4—C5	118.18 (13)	C16—C15—H15A	109.1
C3—C4—C13	121.00 (14)	N4—C15—H15B	109.1
C5—C4—C13	120.81 (14)	C16—C15—H15B	109.1
C6—C5—C4	122.51 (13)	H15A—C15—H15B	107.8
C6—C5—H5A	118.7	C15—C16—H16A	109.5
C4—C5—H5A	118.7	C15—C16—H16B	109.5
C5—C6—C1	119.12 (13)	H16A—C16—H16B	109.5
C5—C6—C14	123.30 (13)	C15—C16—H16C	109.5
C1—C6—C14	117.50 (13)	H16A—C16—H16C	109.5
N1—C7—C12	108.20 (13)	H16B—C16—H16C	109.5
N1—C7—C8	130.99 (15)	N4—C17—C18	113.18 (17)
C12—C7—C8	120.82 (14)	N4—C17—H17A	108.9
C9—C8—C7	116.78 (16)	C18—C17—H17A	108.9
C9—C8—H8A	121.6	N4—C17—H17B	108.9
C7—C8—H8A	121.6	C18—C17—H17B	108.9
C8—C9—C10	122.39 (17)	H17A—C17—H17B	107.8
C8—C9—H9A	118.8	C17—C18—H18D	109.5
C10—C9—H9A	118.8	C17—C18—H18A	109.5
C11—C10—C9	121.82 (16)	H18D—C18—H18A	109.5
C11—C10—H10A	119.1	C17—C18—H18B	109.5
C9—C10—H10A	119.1	H18D—C18—H18B	109.5
C10—C11—C12	116.99 (16)	H18A—C18—H18B	109.5
C10—C11—H11A	121.5		
			
C7—N1—N2—N3	−0.03 (18)	N2—N1—C7—C12	0.14 (17)
C7—N1—N2—C2	178.32 (13)	N2—N1—C7—C8	−179.45 (17)
N1—N2—N3—C12	−0.08 (17)	N1—C7—C8—C9	179.60 (17)
C2—N2—N3—C12	−178.45 (13)	C12—C7—C8—C9	0.1 (2)
O—C1—C2—C3	179.28 (14)	C7—C8—C9—C10	0.5 (3)
C6—C1—C2—C3	1.2 (2)	C8—C9—C10—C11	−0.5 (3)
O—C1—C2—N2	1.3 (2)	C9—C10—C11—C12	−0.1 (3)
C6—C1—C2—N2	−176.75 (13)	N2—N3—C12—C7	0.16 (16)
N3—N2—C2—C3	−5.1 (2)	N2—N3—C12—C11	−179.77 (17)
N1—N2—C2—C3	176.66 (14)	N1—C7—C12—N3	−0.20 (18)
N3—N2—C2—C1	172.94 (14)	C8—C7—C12—N3	179.44 (15)
N1—N2—C2—C1	−5.3 (2)	N1—C7—C12—C11	179.74 (15)
C1—C2—C3—C4	−0.2 (2)	C8—C7—C12—C11	−0.6 (2)
N2—C2—C3—C4	177.75 (14)	C10—C11—C12—N3	−179.46 (17)
C2—C3—C4—C5	−0.8 (2)	C10—C11—C12—C7	0.6 (3)
C2—C3—C4—C13	−179.95 (15)	C17—N4—C14—C6	−84.24 (18)
C3—C4—C5—C6	0.9 (2)	C15—N4—C14—C6	150.36 (14)
C13—C4—C5—C6	−179.94 (15)	C5—C6—C14—N4	−3.9 (2)
C4—C5—C6—C1	0.1 (2)	C1—C6—C14—N4	179.50 (14)
C4—C5—C6—C14	−176.51 (15)	C14—N4—C15—C16	−76.89 (18)
O—C1—C6—C5	−179.32 (14)	C17—N4—C15—C16	158.01 (16)
C2—C1—C6—C5	−1.1 (2)	C14—N4—C17—C18	158.75 (16)
O—C1—C6—C14	−2.6 (2)	C15—N4—C17—C18	−76.0 (2)
C2—C1—C6—C14	175.67 (14)		

### Hydrogen-bond geometry (Å, °)

**Table:**

*D*—H···*A*	*D*—H	H···*A*	*D*···*A*	*D*—H···*A*
O—H0···N1	0.82	1.90	2.621 (2)	146
